# Precise control of pore hydrophilicity enabled by post-synthetic cation exchange in metal–organic frameworks†
†Electronic supplementary information (ESI) available. See DOI: 10.1039/c8sc00112j


**DOI:** 10.1039/c8sc00112j

**Published:** 2018-03-21

**Authors:** Ashley M. Wright, Adam J. Rieth, Sungwoo Yang, Evelyn N. Wang, Mircea Dincă

**Affiliations:** a Department of Chemistry , Massachusetts Institute of Technology , 77 Mass Ave. , Cambridge , Massachusetts 02139 , USA . Email: mdinca@mit.edu; b Department of Mechanical Engineering , Massachusetts Institute of Technology , 77 Mass Ave. , Cambridge , Massachusetts 02139 , USA

## Abstract

The ability to control the relative humidity at which water uptake occurs in a given adsorbent is advantageous, making that material applicable to a variety of different applications. Here, we show that cation exchange in a metal–organic framework allows precise control over the humidity onset of the water uptake step. Controlled incorporation of cobalt in place of zinc produces open metal sites into the cubic triazolate framework MFU-4*l*, and thereby provides access to materials with uptake steps over a 30% relative humidity range. Notably, the MFU-4*l* framework has an extremely high water adsorption capacity of 1.05 g g^–1^, amongst the highest known for porous materials. The total water capacity is independent of the cobalt loading, showing that cation exchange is a viable route to increase the hydrophilicity of metal–organic frameworks without sacrificing capacity.

## Abstract 2

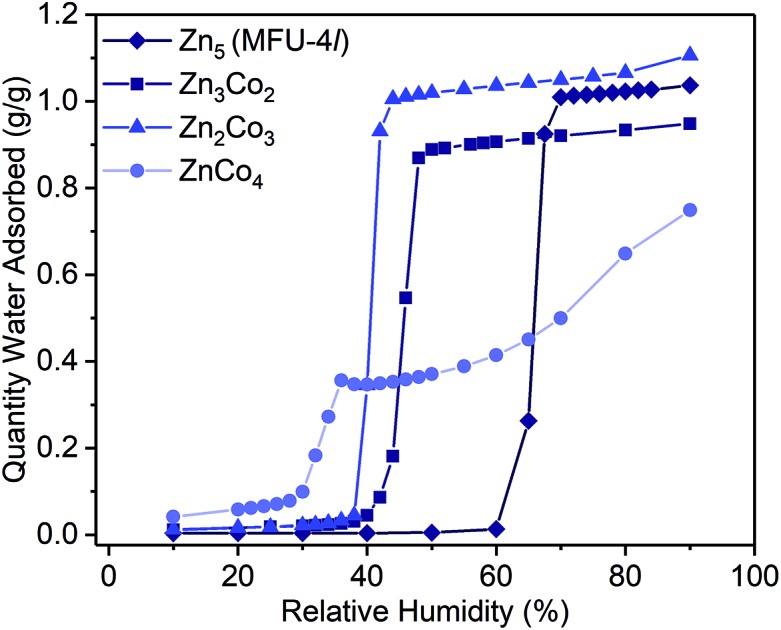
The ability to control the relative humidity at which water uptake occurs in a given adsorbent is advantageous, making that material applicable to a variety of different applications.

## Introduction

Mainstream deployment of devices such as adsorption heat pumps (AHPs) and atmospheric water generators (AWGs) require materials that adsorb water vapor below 100% relative humidity (RH).^1^–^3^ The salient properties of the adsorbent depend on the specific application but generally require water stability, water sorption with a steep uptake step at a desirable relative humidity, and a high total uptake capacity. Metal–organic frameworks (MOFs) have emerged as strong candidate materials for applications involving water vapor sorption due to their record maximal water uptakes, engendered by very high internal void volumes, and tunable material properties. Although many MOFs are unstable to water vapor, MOFs that use azolate linkers such as pyrazoles, imidazoles, and triazoles, exhibit enhanced stability towards polar molecules.^4^–^7^

The functioning parameters of an AHP or an AWG system are determined largely by the position and the steepness of the uptake step, where most of the water is adsorbed. For instance, sorbents that are very hydrophilic may adsorb water at low RH, which is advantageous for applications such as water vapor capture in desert areas, or AHP systems requiring large temperature gradients. However, these advantageous properties come with the caveat that high adsorbent regeneration temperatures are required to remove the water once adsorbed. Control over the position (%RH) of the uptake step is therefore vital to achieve a desirable driving force for water uptake while minimizing the required regeneration temperature.

Due to their modular nature, MOFs are well positioned for precise tuning of the uptake step with regard to both position (*i.e.* RH), and steepness (which can be controlled by modulating the pore size). Control over the position of the uptake step has previously been demonstrated through functionalization of the normally hydrophobic organic linkers.^8^–^11^ However, installation of polar groups on aromatic rings normally results in a reduction of the pore volume, which negatively impacts the overall uptake. An alternative strategy to increase the hydrophilicity of a MOF is to incorporate open metal sites through cation exchange. Cation exchange can be used to functionalize the secondary building units in a variety of MOFs.^12^ By substituting coordinatively saturated metals with cations that can favorably achieve higher coordination numbers, the density of open metal sites where water can bind will increase, consequently increasing hydrophilicity with negligible effect on the pore volume. Herein, we report the first example of water uptake step tuning *via* post-synthetic cation exchange. Increasing substitution of five coordinate-accommodating Co(ii) ions for tetrahedral Zn(ii) in the cubic triazolate framework MFU-4*l* results in precise control of the water uptake step over a range of ∼30% RH.^2^,^6^,^7^

## Results and discussion

**Fig. 1:**
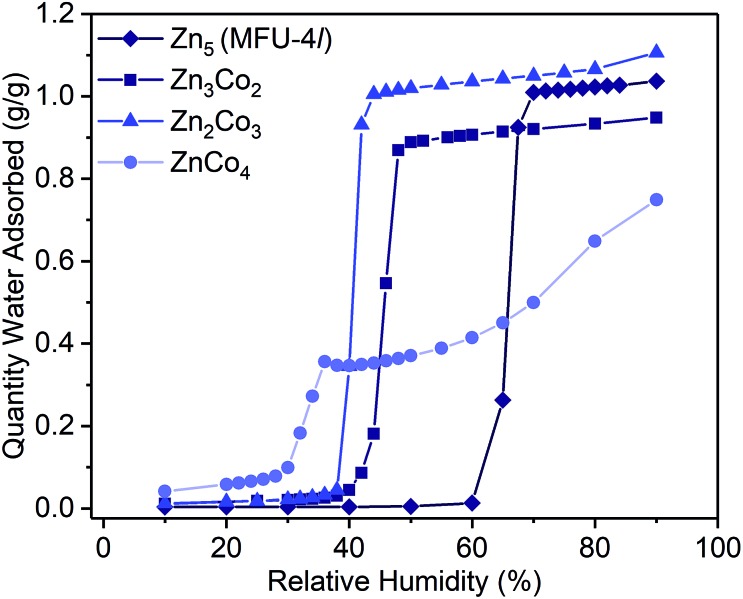
Water adsorption isotherms (298 K) for activated samples of Zn_5_ (MFU-4*l*, **1**, diamonds), Zn_3_Co_2_ (**2**, squares), Zn_2_Co_3_ (**3**, triangles) and ZnCo_4_ (**4**, circles). Symbols are data and the lines are present as a guide for the eye.

Examination of the water sorption properties of MFU-4*l*, Zn_5_Cl_4_(BTDD)_3_ (**1**, BTDD^2–^ = bis(1,2,3-triazolo[4,5-*b*],[4′,5′-i])dibenzo[1,4]dioxin), at 25 °C revealed a type IV isotherm. Very little water is adsorbed between 0–65% RH, but once pore filling is initiated at 65% RH, the uptake step is large and steep with 1.01 g_H_2_O_ g_MOF_^–1^, adsorbed by 70% RH (Fig. 1). The saturation uptake capacity at 95% RH is 1.05 g_H_2_O_ g_MOF_^–1^ indicating most of the water is adsorbed during the sharp uptake step. Notably, the water uptake capacity is amongst the highest reported for any material, surpassed only by MIL-101(Cr) which saturates at 1.3 g_H_2_O_ g_MOF_^–1^.^2^,^8^ Upon water desorption, a small hysteresis loop with a gap of ∼10% RH is observed. For comparison, the isoreticular material MFU-4, which displays a smaller pore adsorbs 0.42 g_H_2_O_ g_MOF_^–1^ with an uptake step between 10% and 35% RH and also exhibits a small hysteresis loop.^13^ Material **1** is stable under water sorption conditions. It retains its crystallinity and porosity as evidenced by N_2_ sorption analysis, which revealed a Brunauer–Emmett–Teller (BET) surface area of 3423 m^2^ g^–1^ after water sorption measurements, in line with the BET surface area of 3525 m^2^ g^–1^ observed for the original MFU-4*l* (Fig. S2†).

**Scheme 1:**
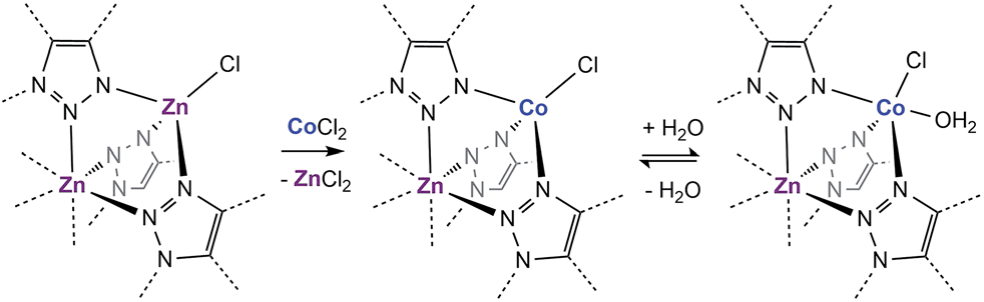
Cation exchange of the peripheral Zn with Co and the subsequent coordination of water in a fragment of the secondary building unit of MFU-4*l*.

With this near-record water saturation capacity, we envisioned that MFU-4*l* could serve as a versatile water sorption material if its hydrophilicity could also be modulated in a systematic manner. To do so, we aimed to take advantage of the metal nodes, where four of the 5 Zn^2+^ ions comprising the secondary building unit (SBU) are peripheral and exposed to the pore surface in a trispyrazolylborate (Tp)-like environment.^14^ Because these Zn^2+^ are tetrahedral and are not known to bind additional solvent molecules, we surmised that replacing zinc ions with Co^2+^ ions, as previously reported, would enable up to four water molecules binding to each SBU (one for each peripheral cation) and potentially enhanced hydrophilicity (Scheme 1).^15^–^20^ Indeed, whereas the majority of reported TpZn compounds are predominately four-coordinate,^21^,^22^ TpCo complexes prefer higher coordination numbers (coordination numbers of 5 and 6 account for >72% of 200 reported structures) with many examples featuring monodentate ligands, such as TpCoCl(THF).^23^

To systematically test the effect of Co^2+^ substitution on the water uptake properties of MFU-4*l*, we prepared materials where, on average, two, three, or all peripheral Zn ions were exchanged with Co^2+^ in each SBU to give Zn_2.9_Co_2.1_Cl_4_(BTDD)_3_ (Zn_3_Co_2_, **2**), Zn_1.8_Co_3.2_Cl_4_(BTDD)_3_ (Zn_2_Co_3_, **3**), and ZnCo_4_Cl_4_(BTDD)_3_ (ZnCo_4_, **4**).^16^ Water adsorption experiments of activated **2** and **3** showed a type IV isotherm with a steep uptake step in a narrow RH range (Fig. 1). Notably, the pore filling step is shifted to lower RH with increasing cobalt loading (*α* values:^24^: **1**, 0.65; **2**, 0.45; **3**, 0.40). Furthermore, the water uptake prior to the pore filling step increased with higher Co loadings, which we attribute to an equilibrium between tetrahedral Co^2+^ and water-bound 5-coordinate Co^2+^. Notably, the amount of water adsorbed prior to the uptake step is approximately one water molecule per cobalt for **2–4**. Adsorption of water prior to the pore filling step enhances pore hydrophilicity, resulting in an onset of capillary condensation at lower RH. Importantly, the total uptake capacity is unaffected by the cobalt loading and ranges between 0.95–1.10 g_H_2_O_ g_MOF_^–1^ for **1–3** (Table 1). Materials **1–3** are stable under the water adsorption conditions and retain crystallinity and porosity after exposure to 0.95 RH as revealed by PXRD (Fig. S1†) and surface area analysis (Fig. S2–4†). Fitting N_2_ adsorption isotherms at 77 K to the BET equation gave apparent surface areas of 2760 and 2799 m^2^ g^–1^ for **2** and **3**, respectively, although lower than the initial values of 3349 and 3543 m^2^ g^–1^ (Fig. S3 and S4†).

**Table 1:** Water adsorption properties of cobalt-exchanged MFU-4*l*

Compound	Zn : Co ratio	Total uptake capacity (g g^–1^)	*α*-value (RH)
**1**	5 : 0	1.04	0.65
**2**	3 : 2	0.95	0.44
**3**	2 : 3	1.11	0.40
**4**	1 : 4	0.75	∼0.30

When all four peripheral Zn atoms are exchanged with Co to give **4**, the framework becomes unstable under measurement conditions. The water adsorption isotherm of **4** features a stepwise adsorption curve. An initial onset of pore filling occurs at 30% RH with a sharp increase until 36% RH when the water capacity reaches 0.35 g_H_2_O_ g_MOF_^–1^ (Fig. 1). Subsequently, a second gradual adsorption occurs with increasing water uptake until saturation at 90% RH, giving a total water uptake of 0.75 g_H_2_O_ g_MOF_^–1^. The isotherm behavior is characteristic of pore collapse at ∼35% RH followed by swelling or condensation of water. Notably, the desorption curve was hysteretic and did not follow the adsorption curve, confirming that a phase change has occurred (Fig. S11†). In agreement with decomposition of the MOF, the surface area after water adsorption was significantly diminished to 680 m^2^ g^–1^, corresponding to a loss of 80% compared to pristine, activated **4** prior to water vapor exposure (Fig. S5†). The phase change observed upon water exposure also occurs with loss of crystallinity, as evidenced by an amorphous PXRD pattern (Fig. S1†).

The water desorption profiles were hysteretic irrespective of the cobalt loading. This is unexpected because hysteresis is generally associated with capillary condensation and is a feature of porous materials with a critical diameter (*D*_c_) greater than 20 Å.^7^ MFU-4*l*, however, is a 3D cubic network with a network of three-dimensionally connected void spaces with pore diameters ranging between 12.0 Å and 18.6 Å. It is particularly noteworthy that the smaller-pore MFU-4 framework also exhibits a slight hysteresis upon an adsorption/desorption cycle of water and its pore sizes are significantly below the *D*_c_.^25^ Alternative reasons for hysteresis in water adsorption by MOFs have been put forward. Recently, the Do–Do model for water adsorption and desorption in activated carbons, has been invoked to explain hysteresis in a microporous MOF.^26^ In this model, water clusters form at sites where water can bind or hydrogen bond until they reach a critical size where they can be sustained within the pore.^27^,^28^ The smaller water clusters subsequently combine to yield larger superclusters, filling the pores in this process. Upon desorption, more energy is required to break up these superclusters back into small water clusters to pass through small pore windows, resulting in hysteresis loops. Therefore, the observed hysteresis may be partially attributed to the topology and pore connectivity of the MOF. In MFU-4*l*, pore windows of only 9.1 Å interconnect larger pore cages. The constricted pore aperture may impede the flow of larger water superclusters that require greater energy to be broken up.

**Fig. 2:**
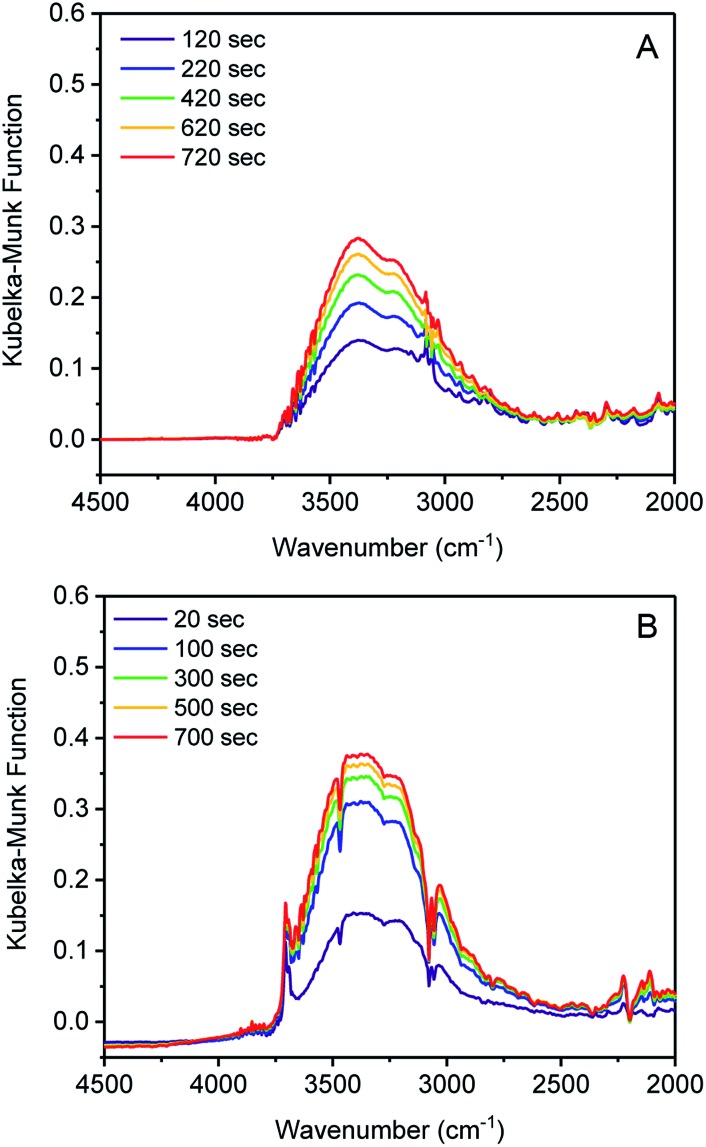
Difference in DRIFTS spectra for the adsorption of water (A) MFU-4*l* (**1**) and (B) Zn_2_Co_3_ (**3**).

Initiation of water clustering can occur by water coordinating to an open metal site or by hydrogen bonding with a polar functional group.^29^ Evidence for water binding to cobalt was obtained by monitoring the adsorption of water in **1** and **3** using diffuse-reflectance IR spectroscopy (DRIFTS). Argon with controlled relative humidity of 80% or 60% was flowed over samples of **1** and **3**, respectively, providing RH levels in slight excess of the position of the water uptake step for both samples. IR spectra were recorded periodically over 20 minutes. Inspection of the difference spectrum for **1** revealed an increase in intensity of two broad bands centered at 3375 and 3200 cm^–1^ assignable to water adsorbed in the pore (Fig. 2A).^29^–^31^ By contrast, when **3** was exposed to water, new bands at 3707 cm^–1^ and 3694 cm^–1^ were initially observed. These remained constant over 1200 seconds (20 min), while the stretching bands of pore-adsorbed water at 3360 and 3210 cm^–1^ continued to grow in intensity (Fig. 2B). An additional band at 1641 cm^–1^ also grows in intensity and is assigned to the bending mode of water (Fig. S12†).^30^,^31^ Bands above 3500 cm^–1^ are assignable to O–H stretches in water with minimal hydrogen bonding.^32^ The stretch at 3707 cm^–1^ is assigned to a cobalt-bound water (Co ← OH_2_) and the peak at 3694 cm^–1^ is assigned to the proximal water molecule hydrogen-bound to the Co ← OH_2_ group (Co ← OH_2_···H_2_O).^32^ Notably, these bands are absent in **1**, which again supports the assertion that water is binding to tetrahedral Co^2+^, but not to tetrahedral Zn^2+^. O–H stretching energies lower than 3500 cm^–1^ are associated with water with many hydrogen bonds, as would be expected for bulk water adsorbed in the pores. Over time the sharp bands at 3707 and 3694 cm^–1^ disappear as the bulk water stretches become much more intense (Fig. S14†). The IR data therefore supports the water clustering mechanism for pore filling, wherein water initially clusters at the cobalt sites and subsequently adsorbs within the pore. When D_2_O was used, the broad feature was shifted to 2520 cm^–1^ and the Co-OD_2_ IR stretch was observed at 2720 cm^–1^. Overall, these results support our hypothesis that water coordination to the open metal center of cobalt is important for lowering the relative humidity/pressure of adsorption.

## Conclusions

The above results illustrate how incorporation of open metal sites through cation exchange provides precise control over the relative pressure of water uptake. The position of the uptake step can be varied over a 30% RH range by controlling the number of sites where water can bind and initiate water clustering, a process which is proposed to play an important role in water adsorption.^24^,^33^ It is also notable that cation exchange does not affect the total water adsorption capacity of the material. The change from zinc to cobalt has a trivial effect on the pore volume and therefore the amount of water adsorbed is within a consistent range for all cobalt loadings. In comparison, alternative methods of increasing pore hydrophilicity such as functionalization with polar organic groups can result in severely reduced uptake capacities. This was the case, for instance, with NH_2_, NO_2_, and SO_3_H derivatives of MIL-101, which adsorb 30–50% less water than their parent material.^8^ These results therefore highlight post-synthetic cation exchange as an alternative method to systematically tune the relative pressure at which water adsorption occurs in MOFs, with cobalt-exchanged MFU-4*l* as an example with near-record, tunable water sorption capacities relevant to water sorption technologies.

## Conflicts of interest

There are no conflicts to declare.

## Supplementary Material

Supplementary information
