# Supplementary material for: Precise control of pore hydrophilicity enabled by post-synthetic cation exchange in metal–organic frameworks
Source: Chem Sci. 2018 Mar 21;9(15):3856–9. doi: 10.1039/c8sc00112j (PMC5941795; doi:10.1039/c8sc00112j)
Supplement: Supplementary file 1 [file SC-009-C8SC00112J-s001.pdf]

Electronic Supplementary Material (ESI) for Chemical Science.
This journal is © The Royal Society of Chemistry 2018

Supporting Information for:

Precise control of pore hydrophilicity enabled by post-synthetic cation exchange in metal-organic frameworks

Ashley M. Wright[1], Adam J. Rieth[1], Sungwoo Yang[2], Evelyn N. Wang[2], Mircea Dincă[1]*

Affiliations:

[1] Department of Chemistry, Massachusetts Institute of Technology, 77 Mass. Ave. Cambridge, Massachusetts, 02139, United States.

[2] Department of Mechanical Engineering, Massachusetts Institute of Technology, 77 Mass. Ave. Cambridge, Massachusetts, 02139, United States.

* Corresponding Author: mdinca@mit.edu

Table of Contents

General Procedures S3
Powder X-ray Diffraction Patterns S5
Nitrogen Isotherms S6
Water Adsorption Isotherms using Dynamic Vapor Source S13
IR spectroscopy........ S17
References S23

General Procedures

Materials

$CoCl_2 \cdot 6H_2O$ (99.9%, Alfa Aesar), $ZnCl_2 \cdot 6H_2O$ (99.9%, Alfa Aesar), HCl (32-35%, BDH – VWR Analytic) methanol (99.9%, VWR), *N,N*-dimethylformamide (DMF, 99.8%, Millipore), ethanol (ACS grade, Mallinckrodt) were used as received. MFU-4*l* was synthesized using previously published procedures.[1] The cobalt-exchanged MOFs were also prepared by a previously reported procedure.[2]

Table S1 shows the materials used in this study with the Zn:Co ratios determined using inductively coupled plasma mass spectrometry (ICP-OES).

Table S1. Comparison of the compound name and number convention with the formula determined from ICP.

Compound Name and Number	Formula based on ICP-OES
Zn_5 (**1**)	$\mathbf{Zn_5}Cl_4(BTDD)_3$
Zn_3Co_2 (**2**)	$\mathbf{Zn_{2.9}Co_{2.1}}Cl_4(BTDD)_3$
Zn_2Co_3 (**3**)	$\mathbf{Zn_{1.8}Co_{3.2}}Cl_4(BTDD)_3$
$ZnCo_4$ (**4**)	$\mathbf{ZnCo_4}Cl_4(BTDD)_3$

Powder X-ray Diffraction (PXRD) patterns were recorded with a Bruker Advance II diffractometer equipped with a θ/2θ Bragg-Brentano geometry and Ni-filtered CuKα radiation ($K\alpha_1$ = 1.5406 Å, $K\alpha_2$ = 1.5444 Å, $K\alpha_1/K\alpha_2$ = 0.5). The tube voltage and current were 40 kV and 40 mA, respectively. Samples for PXRD were prepared by placing a thin layer of the appropriate material on a zero-background silicon crystal plate.

Nitrogen adsorption isotherms were measured by a volumetric method using a Micromeritics ASAP 2020 gas sorption analyzer. Typical samples of ca. 40 mg, preactivated at >100°C to remove all residual solvent, were transferred in an Ar-filled glovebox to a pre-weighed analysis tube. The

tube with sample inside was weighed again to determine the mass of the sample. The tube was capped with a Micromeritics TranSeal™, brought out of the glovebox, and transferred to the analysis port of the gas sorption analyzer. Free space correction measurements were performed using ultra-high purity He gas (UHP grade 5, 99.999% pure). Nitrogen isotherms were measured using UHP grade Nitrogen. All nitrogen analyses were performed using a liquid nitrogen bath at 77 K. Oil-free vacuum pumps were used to prevent contamination of sample or feed gases.

Water adsorption isotherms were measured using Surface Measurement Systems DVS Adventure dynamic gravimetric water sorption analyzer. A typical sample of ca. 5 mg of MOF, pre-activated at 180 °C, but later exposed to air, was loaded into the microbalance. The instrument was set to deliver variable vapor pressures of water corresponding to a relative humidity between 0 and 90%.

In Situ DRIFTS measurements were performed on a Bruker Tensor 37 with a mercury cadmium telluride detector cooled to 77 K. Data were collected in "MIR_DRIFTS" mode with a 6 mm aperture setting and a KBr beam splitter using a DiffusIR accessory made by Pike Technologies in an in-situ cell equipped with a ZnSe window. Data was averaged over 16 scans between 6000 and 600 cm^{-1}. Fully activated samples of MFU-4*l* (**1**) and Zn_2Co_3 (**3**) were loaded in to ceramic sample cup in an argon glove box. A dry argon flow and 'wet' argon flow was attached to mass flow controllers and a T-junction. The humidity of the gas flow was recorded using a VWR humidity detector. An initial spectrum under argon was recorded and then valve was opened to the flow of a specific humidity argon and spectra collection was immediately started and collected at regular intervals thereafter.

Powder X-ray Diffraction Patterns

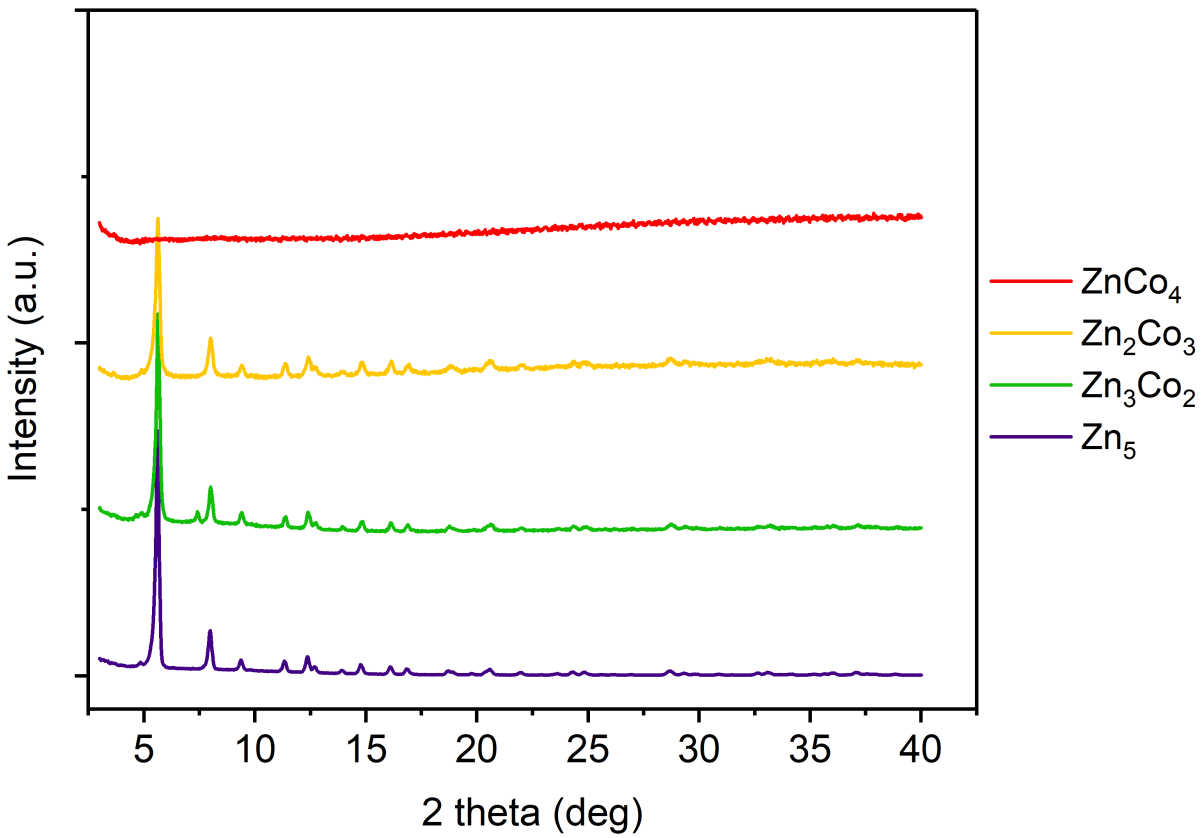

Figure S1. Powder X-ray diffraction patterns of **1–4** after a single water isotherm at 298 K.

Nitrogen Isotherms

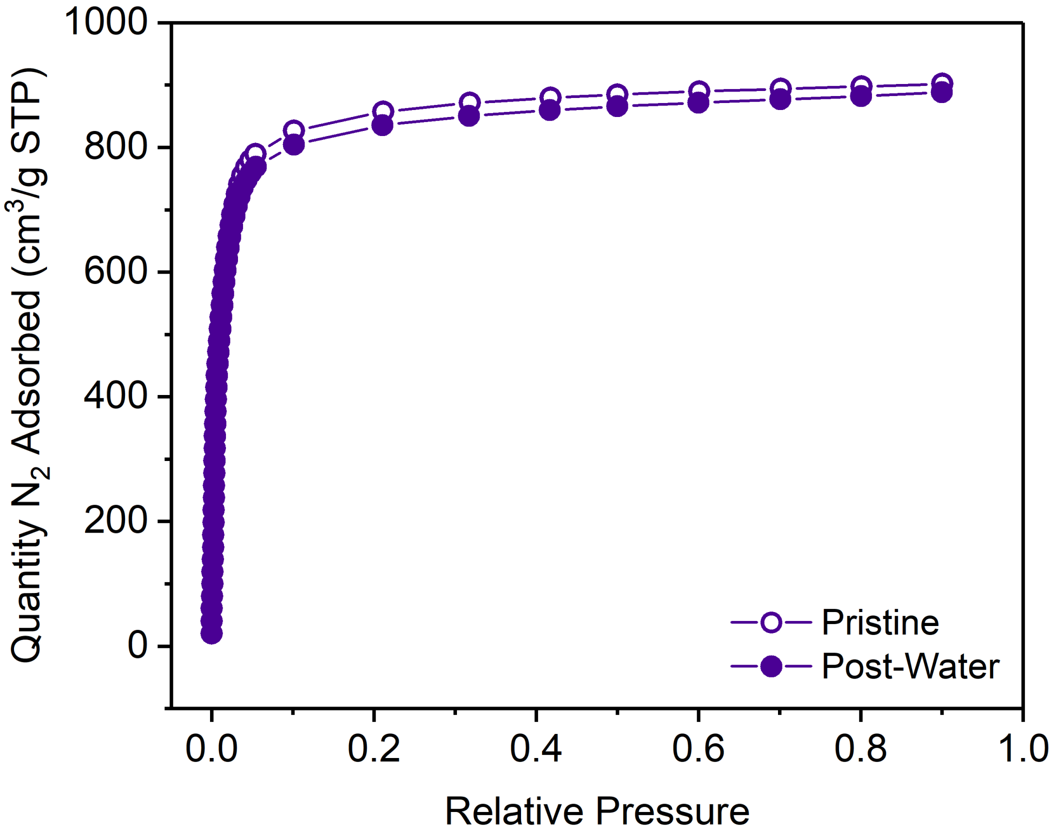

Figure S2. N_2 adsorption isotherms at 77 K of pristine $Zn_5Cl_4(BTDD)_3$ (**1**) (open purple circles) and after reactivation at 150 °C post a single water isotherm at 298 K (closed purple circles). BET surface areas: pristine: 3525 m^2/g, post-water: 3423 m^2/g.

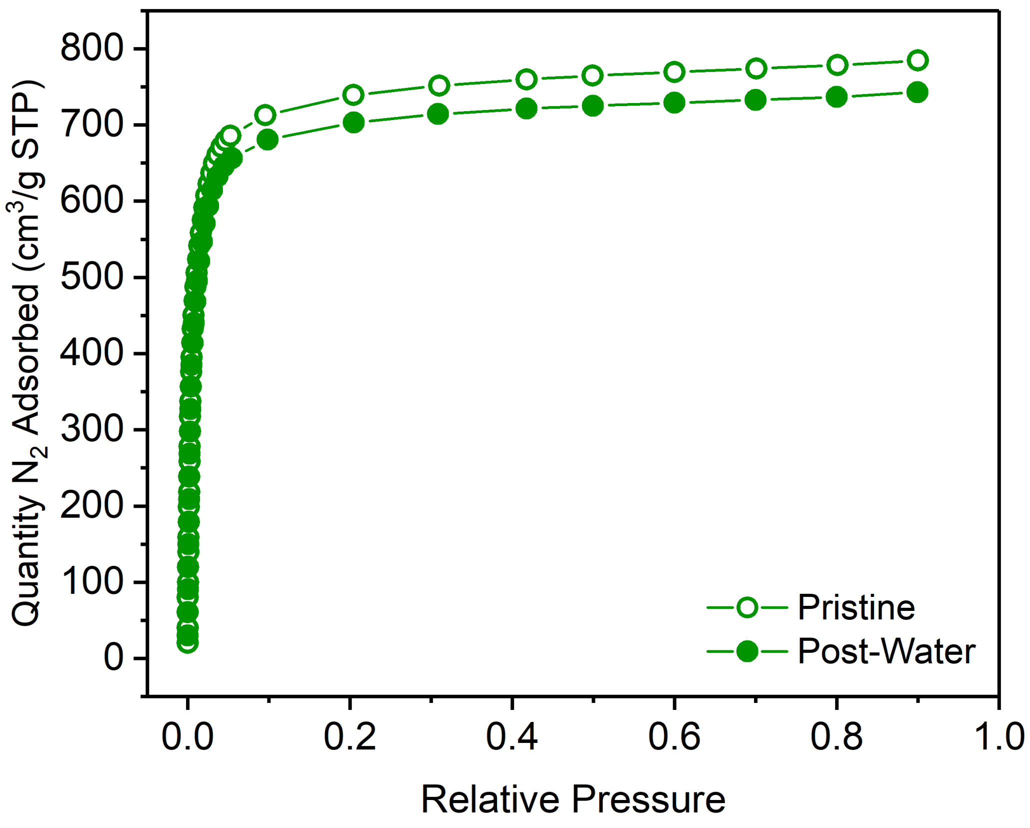

Figure S3. N_2 adsorption isotherms at 77 K of pristine $Zn_3Co_2Cl_4(BTDD)_3$ (**2**) (open green circles) and after reactivation at 150 °C post a single water isotherm at 298 K (closed green circles). BET surface areas: pristine: 3037 m^2/g, post-water: 2760 m^2/g.

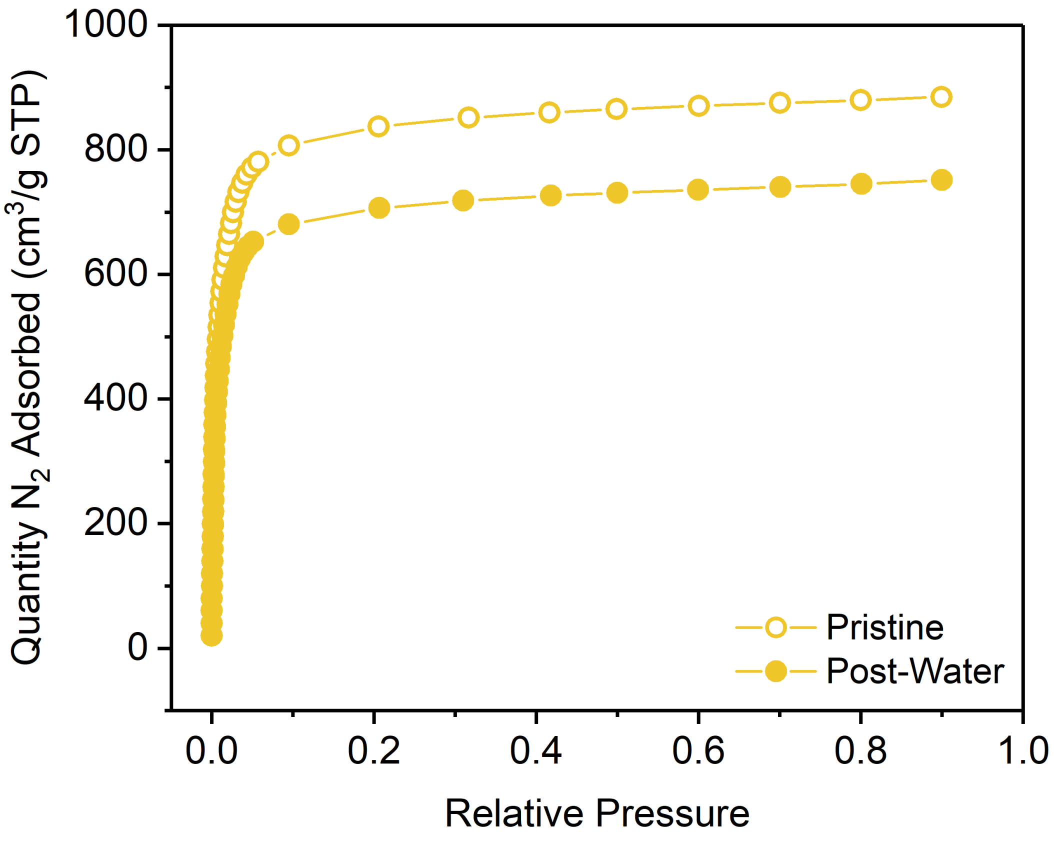

Figure S4. N_2 adsorption isotherms at 77 K of pristine $Zn_2Co_3Cl_4(BTDD)_3$ (**3**) (open gold circles) and after reactivation at 150 °C post a single water isotherm at 298 K (closed gold circles). BET surface areas: pristine: 3544 m^2/g, post-water: 2799 m^2/g.

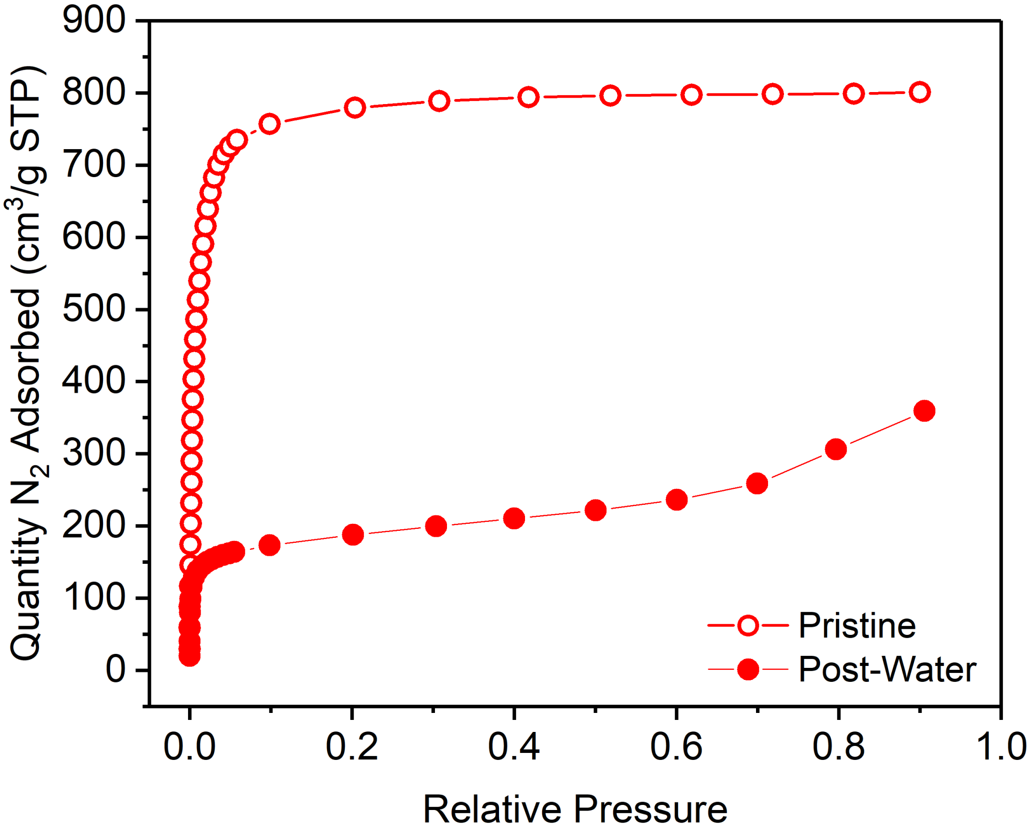

Figure S5. N_2 adsorption isotherms at 77 K of pristine $ZnCo_4Cl_4(BTDD)_3$ (**4**) (open red circles) and after reactivation at 150 °C post a single water isotherm at 298 K (closed red circles). BET surface areas: pristine: 3091 m^2/g, post-water: 680 m^2/g.

Table S1. Comparison of the BET surface area pre- and post-water absorption for the MFU-4*l* and its cobalt exchanged materials.

MOF	BET Surface Area Pre-Water Adsorption (m^2/g)	BET Surface Area Post-Water Adsorption (m^2/g)
Zn_5 (**1**)	3525 ± 65	3423 ± 62
Zn_3Co_2 (**2**)	3037 ± 37	2760 ±26
Zn_2Co_3 (**3**)	3544 ± 21	2799 ±41
$ZnCo_4$ (**4**)	3091 ± 31	680 ± 3

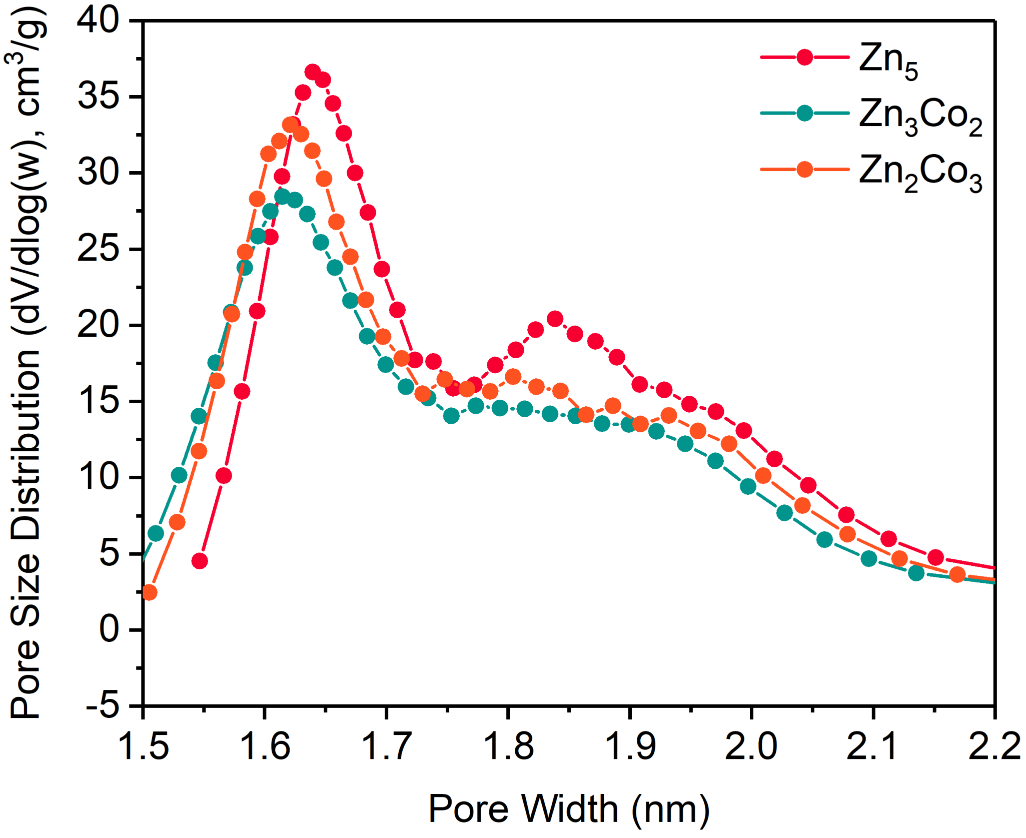

Figure S6. Barrett-Joyner-Halenda (BJH)[3] pore size distribution curves using the Kruk-Jaroniec-Sayari correction. MFU-4*l* (red circles), Zn_3Co_2 (green circles), and Zn_2Co_3 (orange circles). The measured pore widths should not be over interpreted due to incorrect pore geometry considerations.[1] However, the consistent pore width between the three materials suggests cation exchange does not cause significant lattice distortions.

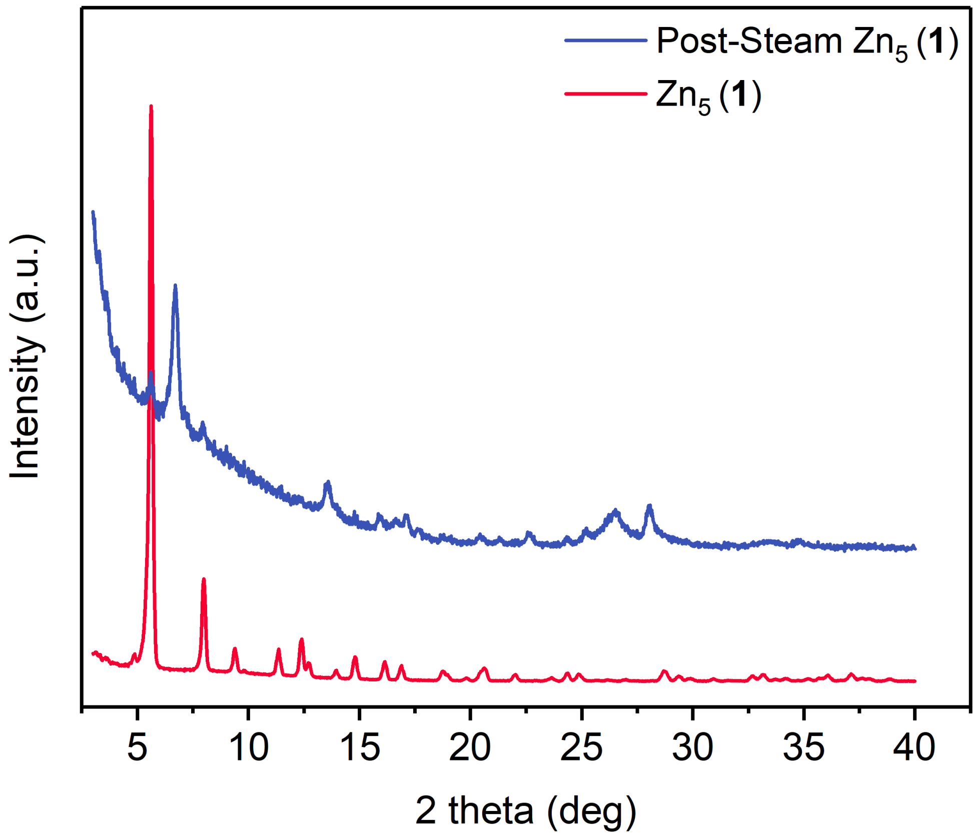

Figure S7. Comparison of the PXRD pattern of MFU-4*l* (**1**) (purple trace) and **1** exposed to steam for 1.5 hours (red trace). The crystallinity of **1** was lost upon exposure to steam.

Water Adsorption Isotherms using Dynamic Vapor Source

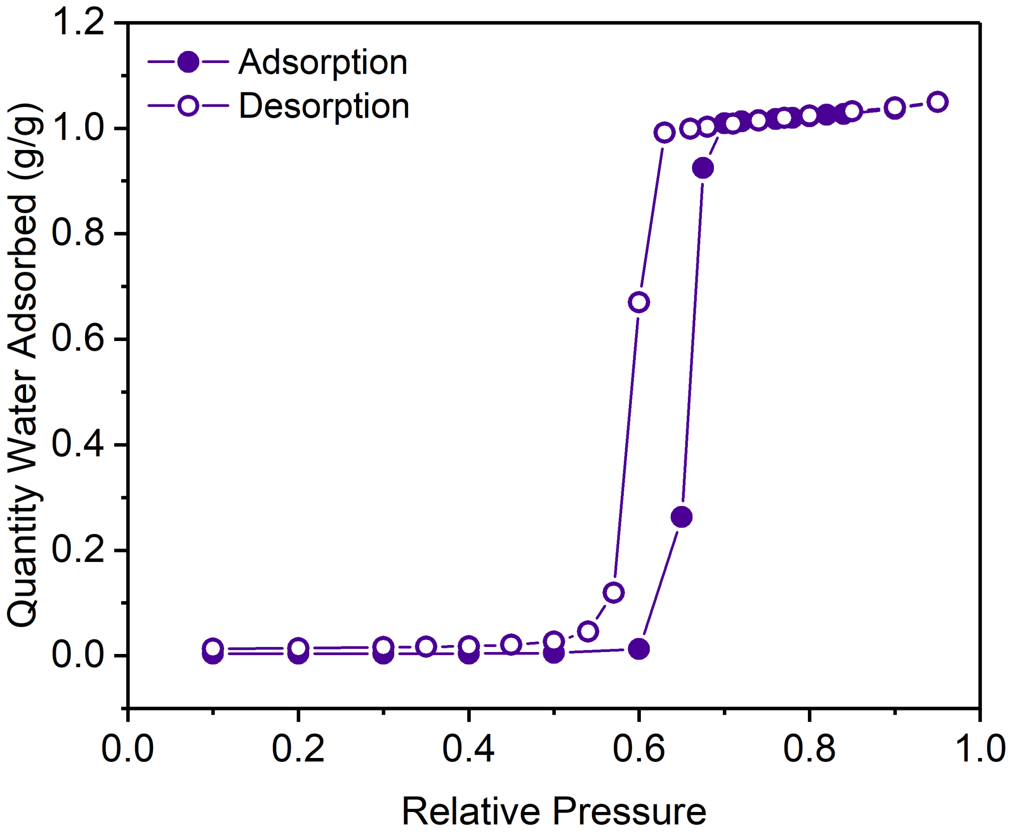

Figure S8. Water adsorption isotherm for Zn_5 (MFU-4*l*, **1**) at 298 K. Closed symbols represent the adsorption and open symbols represent the desorption. Data are symbols and the lines are meant only as a guide for the eye.

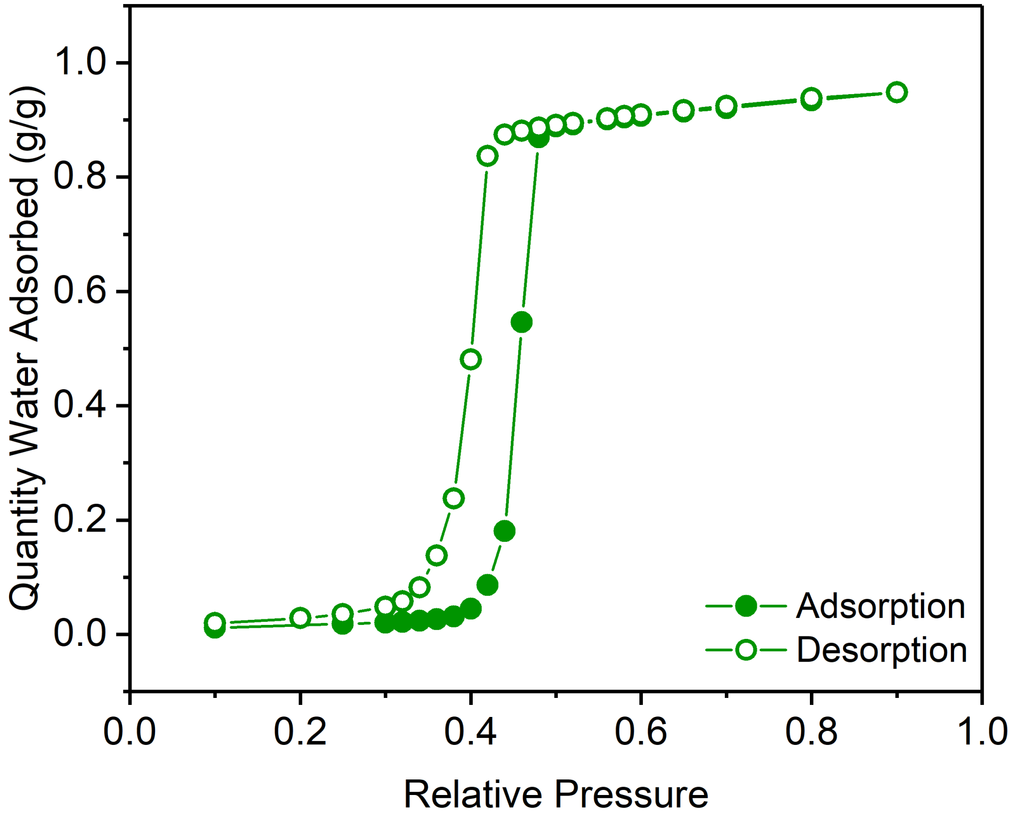

Figure S9. Water adsorption isotherm for Zn_3Co_2 (**2**) at 298 K. Closed symbols represent the adsorption and open symbols represent the desorption. Data are symbols and the lines are meant only as a guide for the eye. The number of water molecules adsorbed per cobalt prior to the uptake step was calculated to be 1.5 at RH 40%.

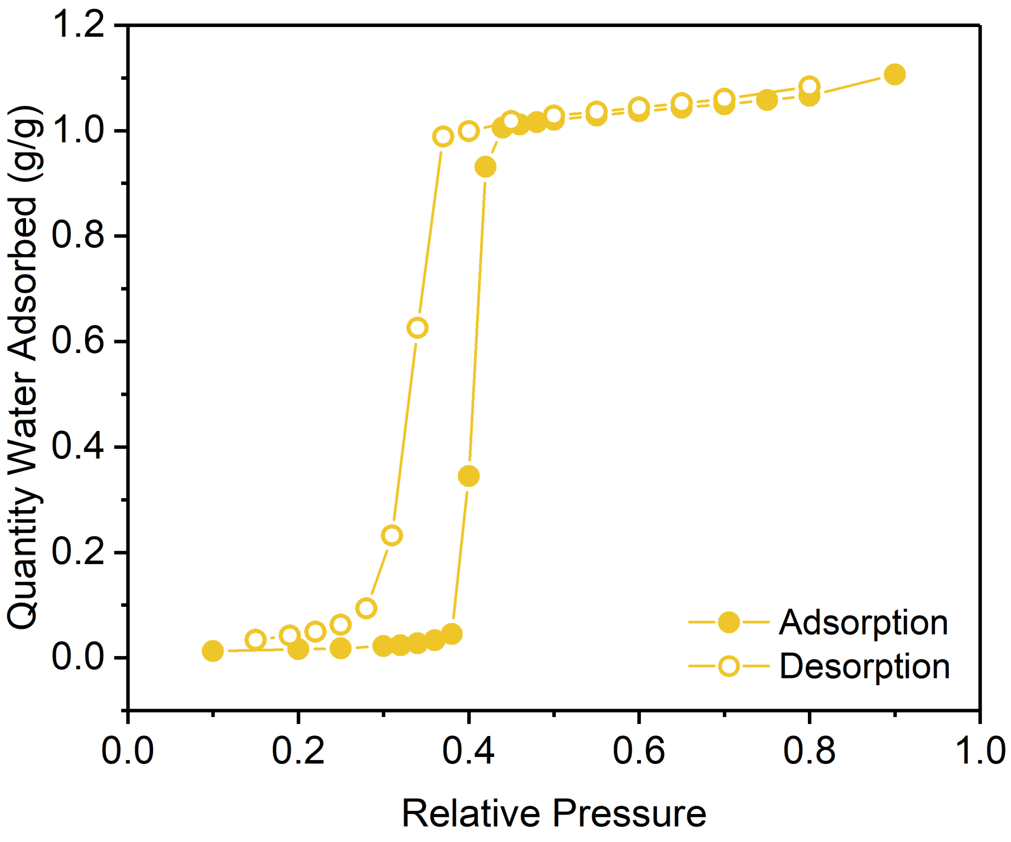

Figure S10. Water adsorption isotherm for Zn_2Co_3 (**3**) at 298 K. Closed symbols represent the adsorption and open symbols represent the desorption. Data are symbols and the lines are meant only as a guide for the eye. The number of water molecules adsorbed per cobalt prior to the uptake step was calculated to be 1.0 at RH 38%.

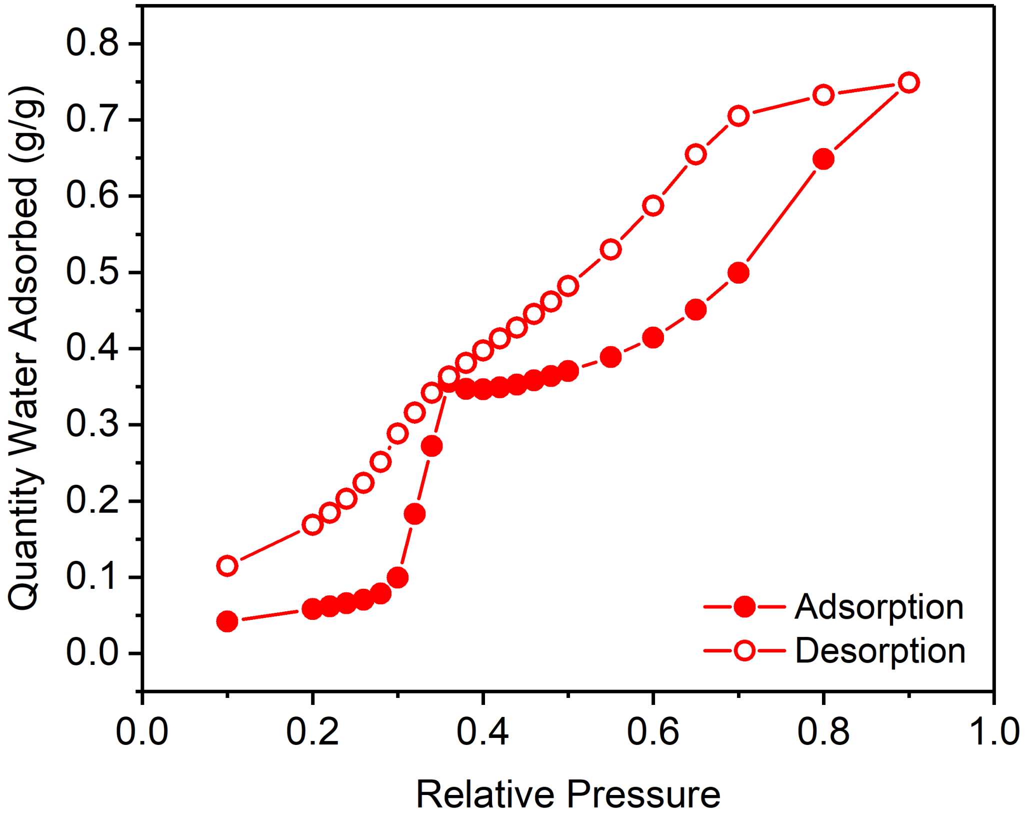

Figure S11. Water adsorption isotherm for $ZnCo_4$ (**4**) at 298 K. Closed symbols represent the adsorption and open symbols represent the desorption. Data are symbols and the lines are meant only as a guide for the eye. The number of water molecules adsorbed per cobalt prior to the uptake step was calculated to be 1.3 at RH 36%.

IR spectroscopy

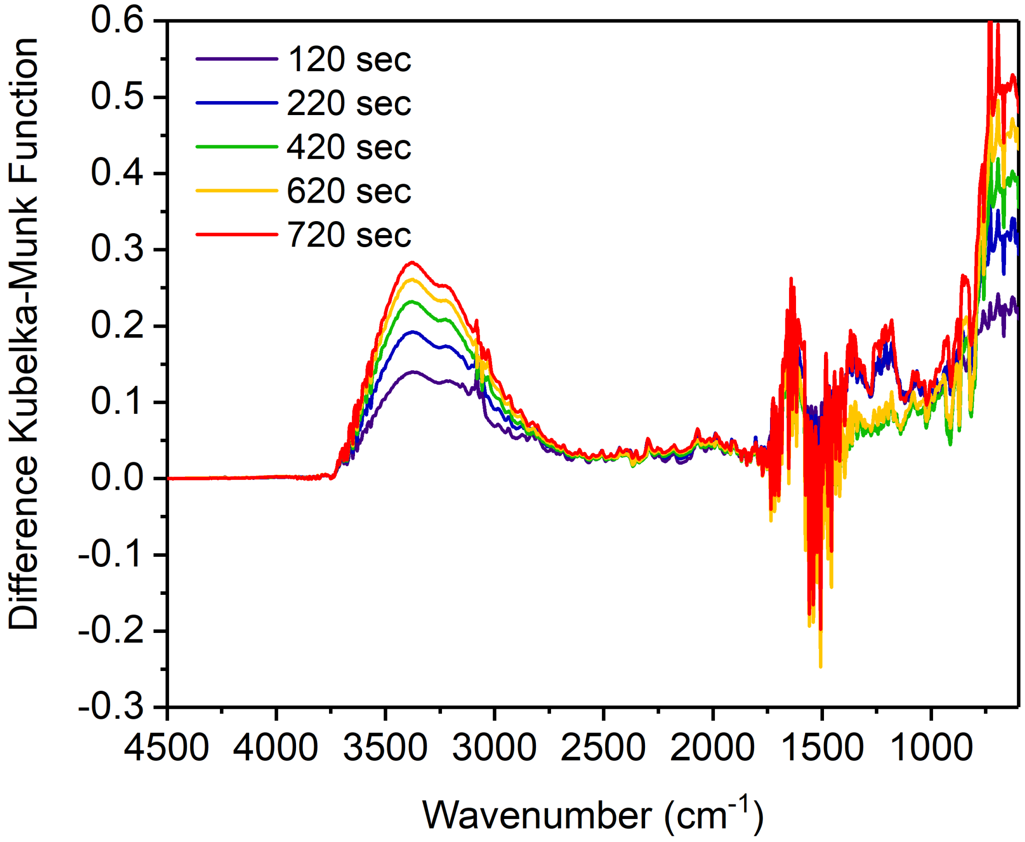

Figure S12. The difference DRIFTS spectra for the adsorption of water by MFU-4*l* (**1**). Monitoring the adsorption of water by MFU-4*l* (**1**) in 80% RH Argon flow.

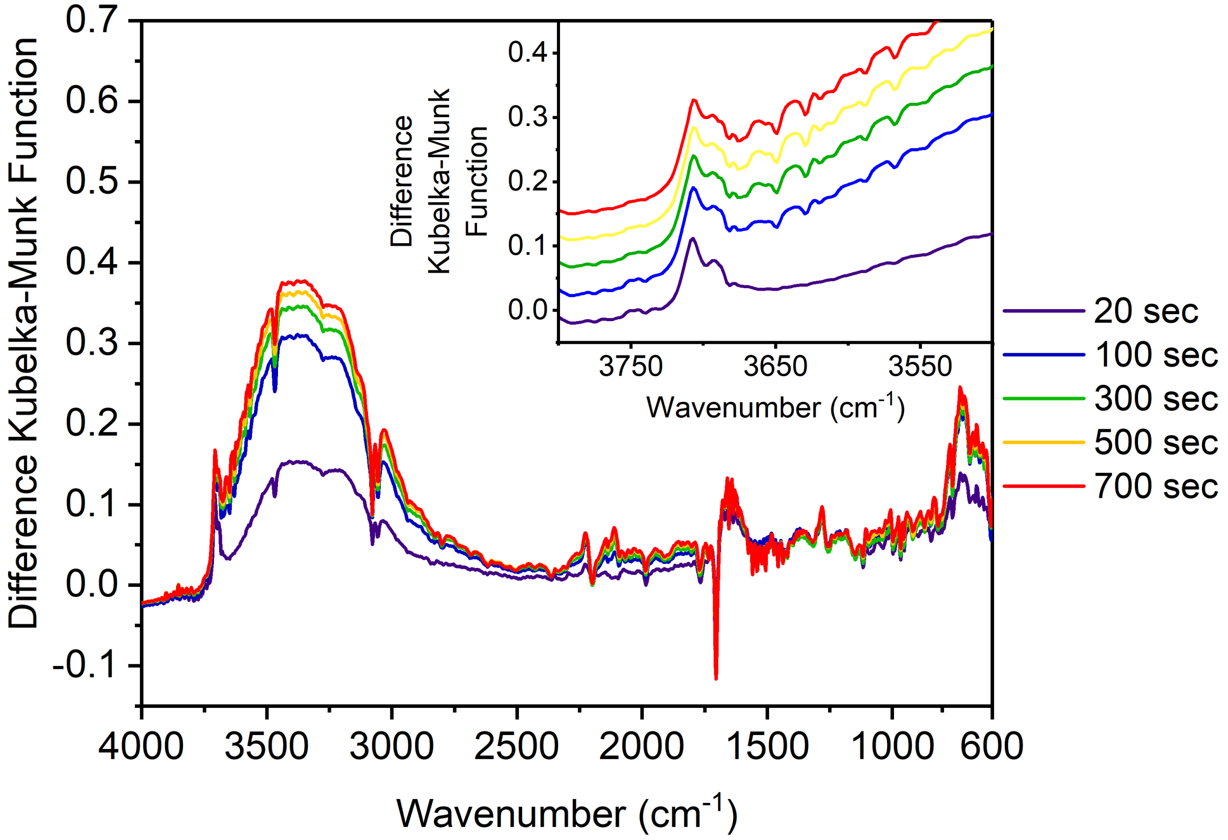

Figure S13. The difference DRIFTS spectra for the adsorption of water by Zn_2Co_3 (**3**). Monitoring the adsorption of water by Zn_2Co_3 (**3**) in 60% RH argon flow.

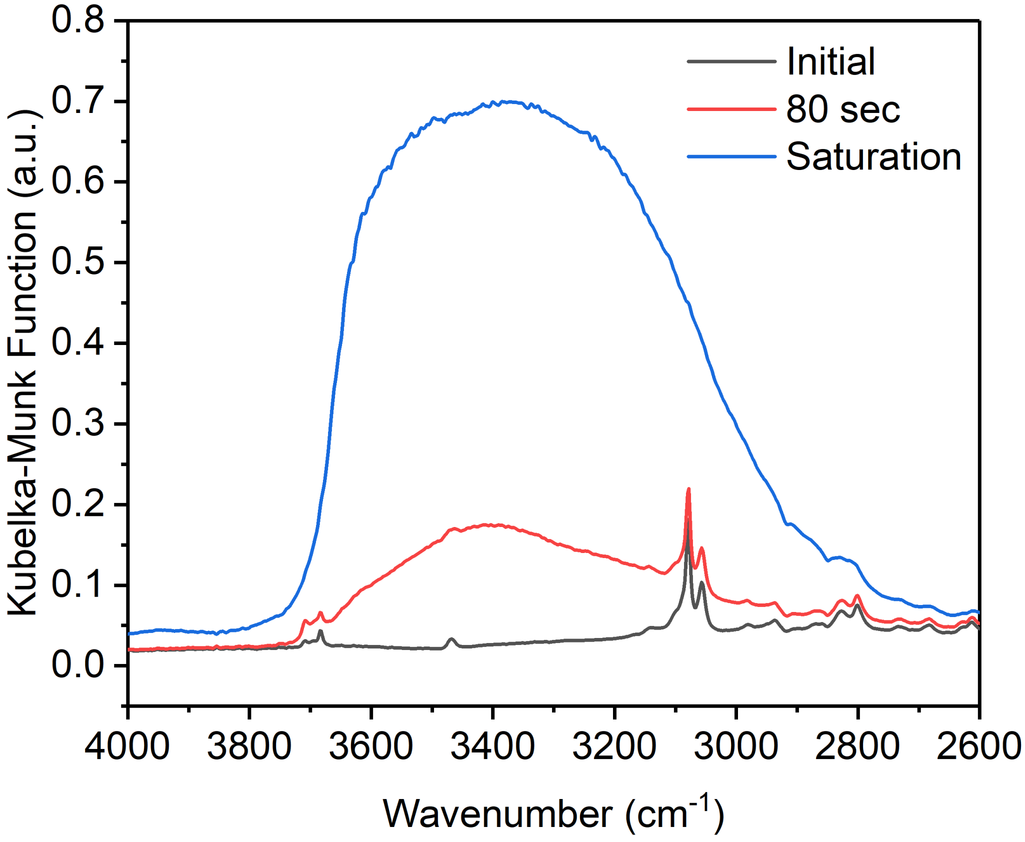

Figure S14. The DRIFTS spectra for the adsorption of water by Zn_2Co_3 (**3**). Monitoring the adsorption of water by 50% RH in argon flow. The grey trace is the initial spectrum. The red trace is after 80 seconds and shows the water adsorbed to the cobalt at 3695 cm^{-1} and water adsorbed within the pore. The blue trace is at saturation and the peak at 3695 cm^{-1} is no longer observed.

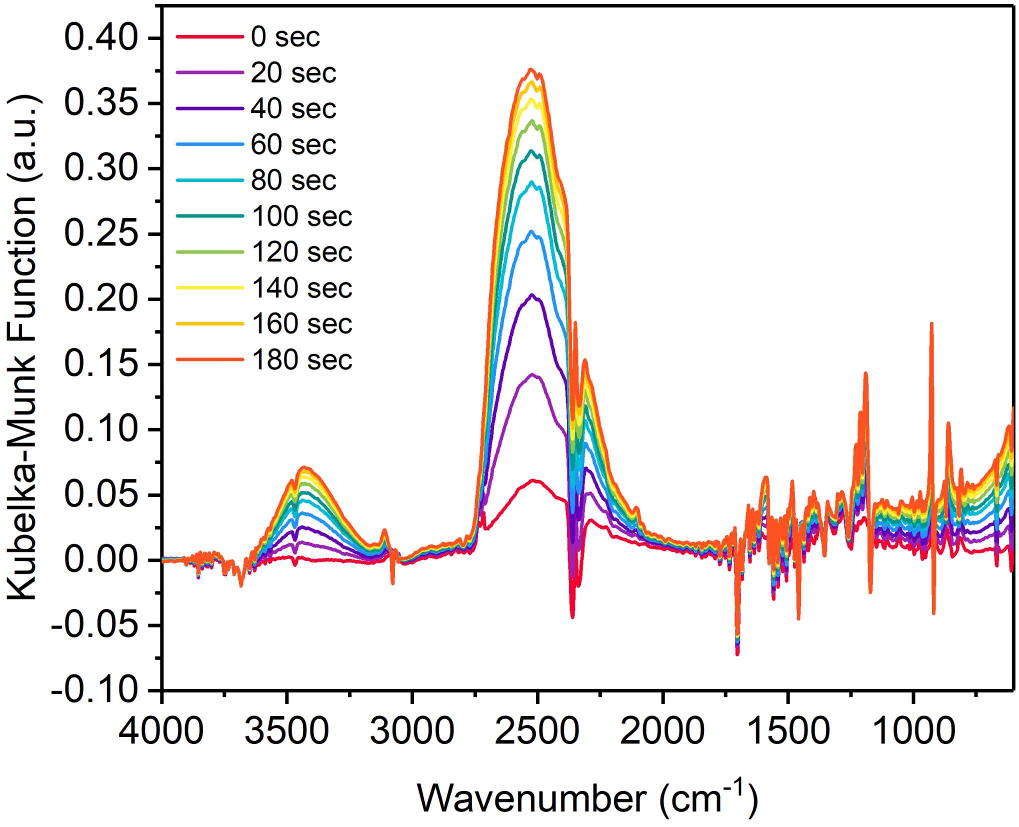

Figure S15a. The difference DRIFTS spectra for the adsorption of D_2O by Zn_2Co_3 (**3**). Monitoring the adsorption of water by Zn_2Co_3 (**3**) in 35% RH Argon flow. The broad feature at 2520 cm^{-1} is assigned to the D_2O within the framework.

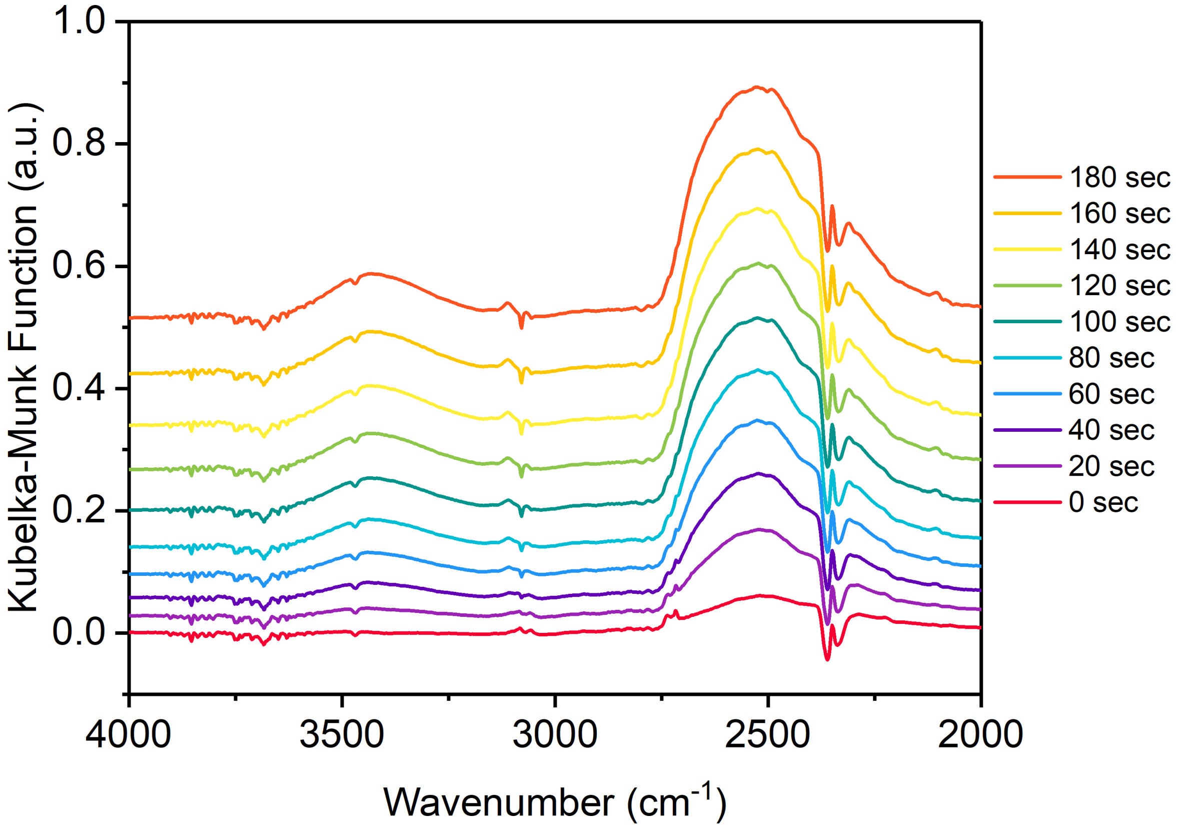

Figure S15b. Stacked difference DRIFTS spectra for the adsorption of D_2O by Zn_2Co_3 (**3**). Monitoring the adsorption of water by Zn_2Co_3 (**3**) with a 35% RH Argon flow. The observed stretch at 2736 cm^{-1} is assigned to the OD stretch of Co←OD_2. The broad feature at 2720 cm^{-1} is assigned to the stretching vibrations of D_2O within the pore of the framework.

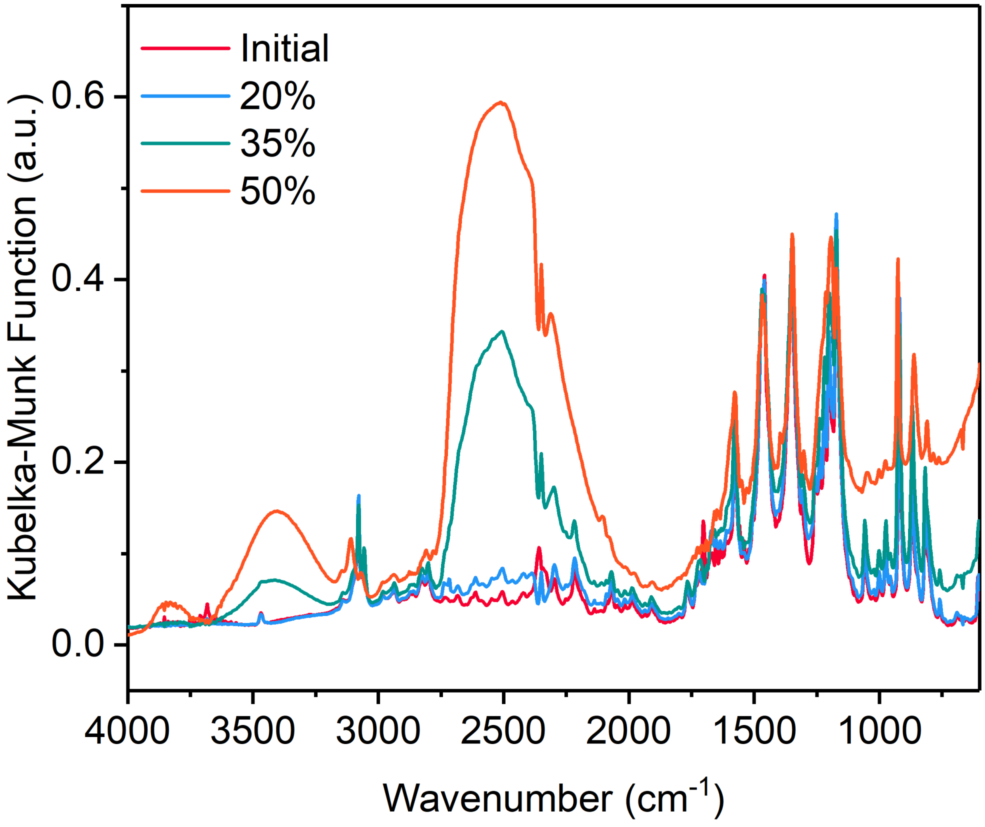

Figure S16. DRIFTS spectra Zn_2Co_3 after exposure to various RH of D_2O. There is a small amount of water in the framework in the initial spectrum at 3685 cm^{-1}, which on addition of D_2O (20% RH, blue spectrum) decreases in intensity. A new signal at 2720 cm^{-1} is observed (blue spectrum), which we have assigned to the new Co←OD$_2$ stretch.

References

(1) Denysenko, D.; Grzywa, M.; Tonigold, M.; Streppel, B.; Krkljus, I.; Hirscher, M.; Mugnaioli, E.; Kolb, U.; Hanss, J.; Volkmer, D. Elucidating Gating Effects for Hydrogen Sorption in MFU-4-Type Triazolate-Based Metal-Organic Frameworks Featuring Different Pore Sizes. *Chem. Eur. J.* **2011**, *17* (6), 1837–1848 DOI: 10.1002/chem.201001872.

(2) Dubey, R.; Comito, R. J.; Wu, Z.; Zhang, G.; Rieth, A. J.; Hendon, C. H.; Miller, J. T.; Dincă, M. Highly Stereoselective Heterogeneous Diene Polymerization by Co-MFU-4l: A Single-Site Catalyst Prepared by Cation Exchange. *J. Am. Chem. Soc.* **2017**, jacs.7b06841 DOI: 10.1021/jacs.7b06841.

(3) Barrett, E. P.; Joyner, L. G.; Halenda, P. P. The Determination of Pore Volume and Area Distributions in Porous Substances. I. Computations from Nitrogen Isotherms. *J. Am. Chem. Soc.* **1951**, *73* (1), 373–380 DOI: 10.1021/ja01145a126.
